# Supplementary material for: Inferring reaction network structure from single-cell, multiplex data, using toric systems theory
Source: PLoS Comput Biol. 2019 Dec 6;15(12):e1007311. doi: 10.1371/journal.pcbi.1007311 (PMC6919632; doi:10.1371/journal.pcbi.1007311)
Supplement: S1 Appendix — (PDF) [file pcbi.1007311.s001.pdf]

S1 Appendix. Steady state analysis of network (3) with unobservables.

This network is deficiency zero, and weakly reversible, so it is complex-balanced. Additionally, there is one connected component of the reaction network, so [1] tells us that at steady state, for every pair of nodes x, y, the ratio of their values is

$$\frac{x}{y} = \frac{K_y}{K_x}\,,$$

where K_x, K_y are defined based on the adjacency matrix of the network. Specifically, given the $n \times n$ weighted adjacency matrix A for the n nodes, where the kinetic constants k_i are the weights, we construct the Laplacian A_k of A, by subtracting the sum of each row from the corresponding entry of the diagonal. Then to determine K_x, we remove the x'th column and row from A_k, denote it $A_{k\backslash x}$, and calculate its determinant up to a sign. Rephrased as a formula:

$$K_x = (-1)^{n+1} \det(A_{k\backslash x})\,.$$

In our example, K_B would be calculated as:

$$A = \begin{bmatrix} 0 & k_1 & 0 \\ 0 & 0 & k_2 \\ k_3 & k_{-2} & 0 \end{bmatrix} \qquad A_k = \begin{bmatrix} -k_1 & k_1 & 0 \\ 0 & -k_2 & k_2 \\ k_3 & k_{-2} & -k_3 - k_{-2} \end{bmatrix}$$

$$K_B = (-1)^{3+1} \det\left(\begin{bmatrix} -k_1 & 0 \\ k_3 & -k_3 - k_{-2} \end{bmatrix}\right) = k_1(k_3 + k_{-2}).$$

The quantities K_A, K_C can be calculated similarly, and their ratios are the constants in the steady state constraints describing (3).

Geometrically, the steady state set $\mathcal{E}$ specified by

$$K_B/K_A \equiv K_1 = [A]/[B] \qquad\qquad K_C/K_A \equiv K_2 = [A]/[C]$$

can be parameterized by t as a line:

$$([A], [B], [C]) = (t, K_1 t, K_2 t).$$

Supposing we only observe A and B, we are left with the line

$$([A], [B]) = (t, K_1 t)$$

which, after taking the logarithms of each coordinate, becomes

$$(\log([A]), \log([B]) = (\log(t), \log(K_1) + \log(t)),$$

or in a different form, taking $T = \log(t)$:

$$(\log([A]), \log([B]) = (0, \log(K_1)) + T(1, 1).$$

The orthogonal complement is spanned by $(1, -1)$, since $(1, -1) \cdot (1, 1) = 0$. This signifies the existence of a net balancing reaction between A and B in the full, unobserved network.

References

1. Craciun G, Dickenstein A, Shiu A, Sturmfels B. Toric dynamical systems. Journal of Symbolic Computation. 2009;44(11):1551–1565. doi:10.1016/j.jsc.2008.08.006.
