# Supplementary material for: Inferring reaction network structure from single-cell, multiplex data, using toric systems theory
Source: PLoS Comput Biol. 2019 Dec 6;15(12):e1007311. doi: 10.1371/journal.pcbi.1007311 (PMC6919632; doi:10.1371/journal.pcbi.1007311)
Supplement: S2 Appendix — (PDF) [file pcbi.1007311.s002.pdf]

S2 Appendix. Probability of manual gap choice producing observed vector entry distribution

In the FACS dataset, choosing a gap after k eigenvalues gives k eigenvectors each with $d = 11$ entries. Some of these dk entries will be forced to 1 or 0 after row reduction, and so only $(d-k)k$ entries are independent. In our data, we typically chose $k = 7$, leading to 28 independent entries. In the worst case, we could choose $k = d - 1$, leading to 10 free entries. Consider the observation that the distribution of entries was almost entirely negative or 0. For one condition, the probability of a given gap choice producing that asymmetry for a random set of vectors is at most $\sim (\frac{1}{2})^{10}$, and the chance that at least one of the 10 gap choices gives the asymmetry is $\sim 2 \times 10^{-3}$.

However, suppose that we include sparseness of the vectors in our null to match the observed vectors' sparseness, leading to a gap that includes k eigenvalues to specify $\sim k$ nonzero, independent entries that are equally likely to be positive or negative. Most of our gaps had at least $k > 5$. The probability of k entries all being negative is $\sim (\frac{1}{2})^k$, so that for a random dataset with sparse structure, the chance of any such gap existing is $\sum_{k=6}^{10} (\frac{1}{2})^k \sim 0.03$. Pessimistically assuming that all 13 samples had identical structure, 0.03 is the probability that a random, sparse structure would even admit a gap choice that produces the observed asymmetry. This probability decreases further if we had lower sparsity, or if we account for the 13 samples having at least partially different structure due to the difference in perturbation conditions between them.

Additionally, our entry distribution showed peaks. The probability of the observed peaks' prominence, p_{peak}, is independent of the probability of asymmetry, although harder to estimate. Therefore, the probability that a dataset would even admit gap choices that produce the key features of our entry distribution is $< (0.03)p_{\text{peak}}$.
