# Supplementary figures and images for: Inferring reaction network structure from single-cell, multiplex data, using toric systems theory
Source: PLoS Comput Biol. 2019 Dec 6;15(12):e1007311. doi: 10.1371/journal.pcbi.1007311 (PMC6919632; doi:10.1371/journal.pcbi.1007311)

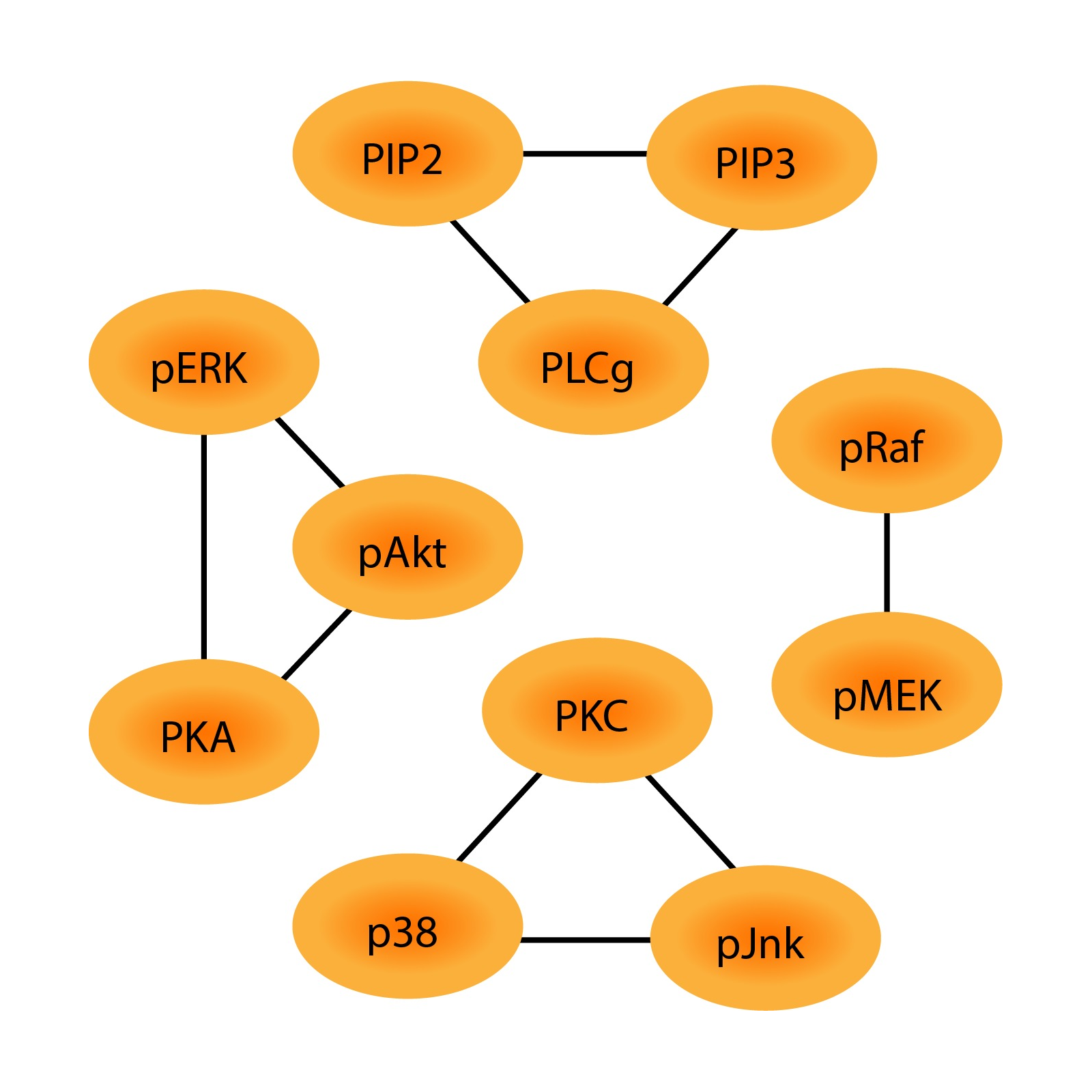

Supplement: S1 Fig — Given recombined, singular vectors for the condition of activation with anti-CD3, anti-CD8 and inhibition of Protein Kinase C with G06976, we drew an edge between biomarkers if any vector entries had magnitude larger than 0.2. (TIF) [file pcbi.1007311.s003.tif]
