# Supplementary material for: Inferring reaction network structure from single-cell, multiplex data, using toric systems theory
Source: PLoS Comput Biol. 2019 Dec 6;15(12):e1007311. doi: 10.1371/journal.pcbi.1007311 (PMC6919632; doi:10.1371/journal.pcbi.1007311)
Supplement: Attachment — Submitted filename: Response to Reviewers.docx [file pcbi.1007311.s005.docx]

**Detailed Response to Review**

Review: Inferring reaction network structure from single-cell, multiplex data, using toric systems theory, PCOMPBIOL-D-19-01320.

**Reviewer #1:**
I find this article a nice contribution to the literature, it proposes a novel combination of network structure and statistical study of reaction networks. Partial linearities in log coordinates of steady states of the system are exploited. I suggest acceptance after the authors incorporate my comments below in a revised version of their submission.

**1)** The stoichiometric subspace S is defined as the linear span of the reaction vectors on line 55, which is correct. However, the reaction vector v defined on line 47 is not the true reaction vector whenever some of the reactant R_i coincides with a product P_j, This does not necessarily happen, e.g. this is not the case in the example on line 198. So this is misleading and it becomes an obscure concept for the reader, while it is very easy to compute S if the whole reaction network is known. Please, define S clearly and explain that its computation is straightforward if the network is known.

*(l.51-55, l.65) We agree that our definition of the reaction vector on line 47 is erroneous,* *and the definition of the reaction vector has therefore been corrected.*

*We now also emphasize S’s computation is straightforward, and point to the examples in the paper. In the definition of S on line 55 we now write: “S, defined as the linear span of all the reaction vectors [27], which is a simple calculation for any given network. In the example…”*

**2)** In display (3), it would be better to add the arrow from C to A curved back to the first node A on the left instead of adding a new node A. This weak reversibility is what allows for a complex balanced steady state and this is an equivalent condition for a linear network as this one.

*(l.92) As suggested by the reviewer, the network figure has been changed to a triangular loop.*

**3)** On line 214 it seems that you are missing some references, e,g: SIAM J. Appl. Dyn. Syst., 17(2), 1650–1682. The Structure of MESSI Biological Systems. Mercedes Pérez Millán and Alicia Dickenstein. https://doi.org/10.1137/17M1113722
Please, take a look at the notion of toric MESSI systems and how the networks are amenable to analytical treatment without simulations (as in several other recent articles). Another important missing reference is:J Theor Biol. 2009 Dec 21;261(4):626-36. The rational parameterization theorem for multisite post-translational modification systems. Thomson M1, Gunawardena J. doi: 10.1016/j.jtbi.2009.09.003.

*(l.260-263) We thank the reviewer for the suggestion of additional references – we have included them in the revised text. We have also changed the text to correct the inaccuracy pointed out by the reviewer with respect to analytical treatment of reaction networks. The text now reads: “Although some classes of non-complex-balanced have been treated analytically [31,36-38], we relied on simulation to study non-toric reaction networks in the context of sc-data.”*

**4)** Please add clearly in all your figures (and their corresponding explanations) which are the values of the reaction rate constants that are being taken into account.

*(l. 68, 74-75, l. 174, 181-182, l.279 & l.281-282) Thank you for pointing out this oversight. For figures 1 and 2, we have directly added the kinetic constants to the figure’s captions, as well as the main body text. For Figure 3, we have included a zip-file containing the weighted adjacency matrices of the reaction networks underlying the simulations, as well as the ordered list of the complex nodes of the networks in the supplementary info.*

**5)** On line 232, explain which is the assumption needed in order that the consideration of a single protein production step is sensible.

*(l.283-284) We have included a sentence to describe the assumption: “The interpretation of this assumption is that mRNA turnover is faster than protein turnover, which is not biologically unreasonable [40].”*

**6)** Please, make the definition of EES more visible, add a pointer to it from the very beginning of the paper (and consider giving it before in the text).

*(l.226-227, 119-122) We have renamed the relevant subsection to “Networks other than those with complex balance may still have toric geometry: the Effective Stoichiometric Space.” Also, in the Overview subsection of Results, we introduce the ESS early: “Exploring non-complex-balanced networks by simulation and examples, we find that our analysis method still recovers subspaces tied to network topology, analogous to how $S$ is tied to reaction vectors. We call these data-derived subspaces Effective Stoichiometric Spaces (ESS), with the precise definition given below.”*

**7)** May be it is useful to take a look at section 5 of the MESSI reference above: when the positive steady states can be defined completely by binomial equations (linear after taking logarithms), the comparison of the matrix built from the exponents of these binomials and a matrix given the linear conservation relations defining S, can be used to detect multistationarity. In this case, the analysis cannot be done for all choices of parameters at the same time, but in regions of mono or multistationarity separately (plus finer stability considerations).

*(l. 528) The reference suggested by the reviewer provides interesting ideas that we hope to use in future work. Unfortunately, in the context of the method we present here, and the single-cell data available to us, we are not able to determine conservation relations, and thereby leverage the concepts suggested by the reviewer. However, in the Discussion, we have explicitly mentioned the potential use of MESSI results to study multistability alongside toric steady states: “Multistable, toric steady state sets exist, with many biologically relevant ones studied recently in the class of toric MESSI systems [30]”.*

**8)** Your example on page 12, line 344 is easily seen to be s-toric MESSi. You are missing the fact that d[D]/dt = - k_d [S][D] – k_{-d} [SD], thus adding this to d[S]/dt and equating to 0, one immediately gets two binomials: this new one and the previous one involving [E],[S],[F],[P] describing $E$! (without any limit!). By the way, it is unfortunate that the steady state set is denoted by $E$ while E denotes a chemical species. I’d suggest to change the letter for the steady state set.

*(l.416-424) Thank you for pointing out this oversight. Unfortunately, responding to the correction decreases the value of our toy example as a means to explain how drugs might affect an experimentally observed ESS. We have replaced the example with a different one that is only log-linear in various limits.*

*We have also changed the letter used to denote a steady state set to a script-E, to avoid the confusion pointed out by the reviewer.*

**Reviewer #2: The paper discusses an important question: given measurements of a set of chemical species, which reaction network can reproduce the measurements sufficiently well. The authors suggest a novel approach that requires single cell data and that the data indicates that the measurements approach a steady state. The idea is to analyze the spectrum of the covariance matrix of the measurements and look for a "gap", indicating a subset of eigenvalues that can be considered zero. The eigenvectors corresponding to the nonzero eigenvalues are then interpreted as "effective reactions" that span an effective stoichiometric space. This space is interpreted as the image of a matrix that parametrizes the positive real part of the steady state variety.**

**I have only one major concern with the paper, it is not precise enough when it comes to the claim that a reaction network is inferred form the data. Only if one assumes that the system is complex balanced, is the effective stoichiometric space related to the reaction network: steady states of complex balanced systems are parameterized by the orthogonal complement of the stoichiometric space. Hence only in this case one may actually claim to have inferred a reaction network. If the system is not complex balanced, then the relation between the reaction network and the matrix that parametrizes the positive real part of the variety is more involved. I think the authors should describe this more clearly: if one chooses to interpret what is inferred as a reaction network, then the corresponding system is complex balanced (with the all the constraints that imposes on the rate constants). If one does not want to impose complex balance, then one infers a monomial parametrization of the positive real part of the variety (which is remarkable enough).**

*The reviewer makes several excellent points. We also wish to avoid the misconception that the ESS directly infers a reaction network, hence our use of imprecise, broader terms such as “structure” or “guiding reconstruction” to convey that we still infer topological/parameter-independent features of the reaction network . We have made the following changes to clarify this distinction further:*

- *(l.71-74) In the introduction, we have clarified that we do not simply infer S, but that in general we infer analogous notions. Following the reviewer’s suggestion, we also clarify that the specific object inferred depends on what assumptions are made about the network: “In our studies, E will be determined from experimental data, and we will be interested in reconstructing $S$, parts of $S$, or other subspaces of a similar stoichiometric nature; which of these is reconstructed depends on what class of network is assumed to generate the data.”*
- *(l.124-127) In the Overview of Results, we have weakened the qualification that for non-complex-balanced networks, the relation between the parameterization and the network topology is “straightforward”: “…still have steady state sets contained in toric manifolds (either exactly or approximately), whose orthogonal complements in log-concentration space are related to reaction network topology (i.e. independent of kinetic parameters)[28-30].”*
- *In defining the ESS, we have added further explanation (see response to “A suggestion” for details)*

*Regarding the specific point about what can be inferred without imposing complex balance: although we do not recover the traditional stoichiometric subspace, we do not want to ignore the recently described connections between monomial steady-state parameterizations and other notions of reaction topology [29-31]. The edits described above therefore attempt to make a slightly less restricted claim about what is inferred in the non-complex-balanced case: instead of just a “monomial parameterization”, generally, we infer some kind of reaction network topological feature.*

**A suggestion:** the approach can be applied to any data set coming from single cell measurement. If one finds a gap in the covariance matrix, then this indicates that the data can be described by a model that has a remarkable property: the positive real part of the variety can be parameterized by monomials. For generic polynomials this is rarely the case, so maybe one could advertise this.

*(l.313-320) Thank you for this excellent insight and suggestion. We now include this idea when we define the ESS: “In the remainder of this paper we refer to the orthogonal complement of the minimal, linear set containing the log steady state as the effective stoichiomietric space (ESS). The previous examples demonstrate the potential value of the ESS for mechanistic analysis of biological systems, which may often be considered non-complex-balanced. At the very least, if one wants to constrain an asymptotically stable reaction network model using single-cell, multiplex data, a data-derived ESS identifies log-linear relations that must appear in the model's dynamical equations; this is a fairly precise constraint, since generic polynomial equations are seldom log-linear.”*

Some minor points:

**1)** the caption of figure one: I think it should be steady state set, not steady state

*The mistake has been corrected*

**2)** line 67: I think the name is Kirchhoff

*The mistake has been corrected*

**3)** line 404: I do not think that log-linear manifolds arise from mass action kinetics. They require a special network structure, not every mass action network gives rise to such a manifold.

*(l.480-482) The reviewer is correct and our statement was imprecise. We have therefore changed it to make clear that log-linear manifolds arise from both assuming mass-action kinetics AND being within a class of network: “These features of the ESS approach are a direct consequence of toric (log-linear) manifolds that arise from an assumption of mass-action kinetics applicable - at least approximately - to a broad class of networks,”*

**4)** line 406-408. This sentence does not make any sense to me. You say you do not need QSS but that the steady state is asymptotically stable. And then you say that enough time should have passed to approach the QSS What is it that you want to say?

*(l.483-489) We have changed the sentences in this section to clear-up our confusing language****,*** *by changing “approach” to “reach the exponentially stable neighborhood”: “… and hold even under the looser requirement that a steady state is a subset of an approximately toric manifold. If the steady state set fulfills this requirement, the ESS also requires that the steady states be asymptotically stable, and that single cells reach the exponentially stable neighborhood of these steady states. This is looser than requiring that cells be at steady state or quasi-steady state, further expanding the situations in which the ESS framework is informative and applicable.”*

**5)** line 482 what does it mean when you say all trajectories within a coset are forward invariant? It is my understanding that the coset is forward invariant.

*(l.562-563) We thank the reviewer for pointing out our poor word use. The statement has been changed to: “all trajectories of a complex-balanced system remain within the coset, by forward-invariance of these cosets”*

**6)** line 488 you could state that the upper bound is for all trajectories within a coset, just to be precise.

*(l.575-576) We have added the suggested language. The relevant revised sentence reads: “upper bound on the distance between any pair of trajectories in a coset. As distance between these trajectories decreases, their variance decreases.*

**7)** reference 18. As far as I know, the authors describing mass action kinetics for the first time are called Waage and Guldberg.

*This mistake has been corrected*
